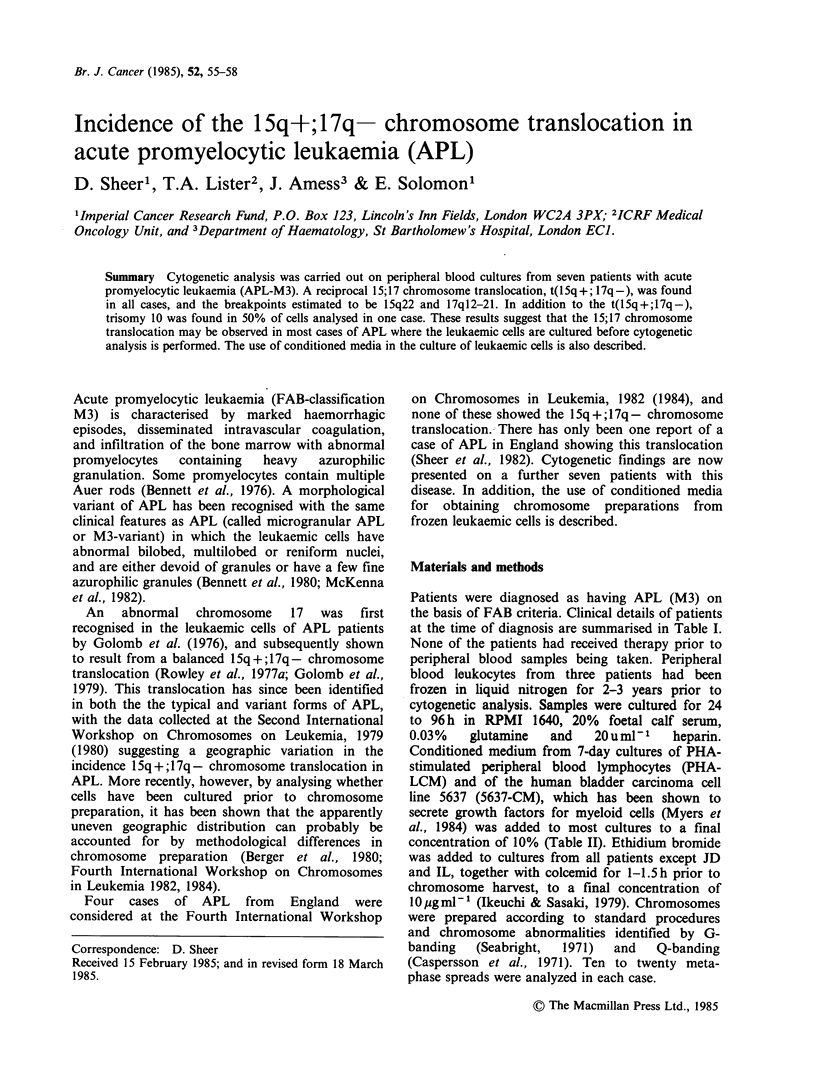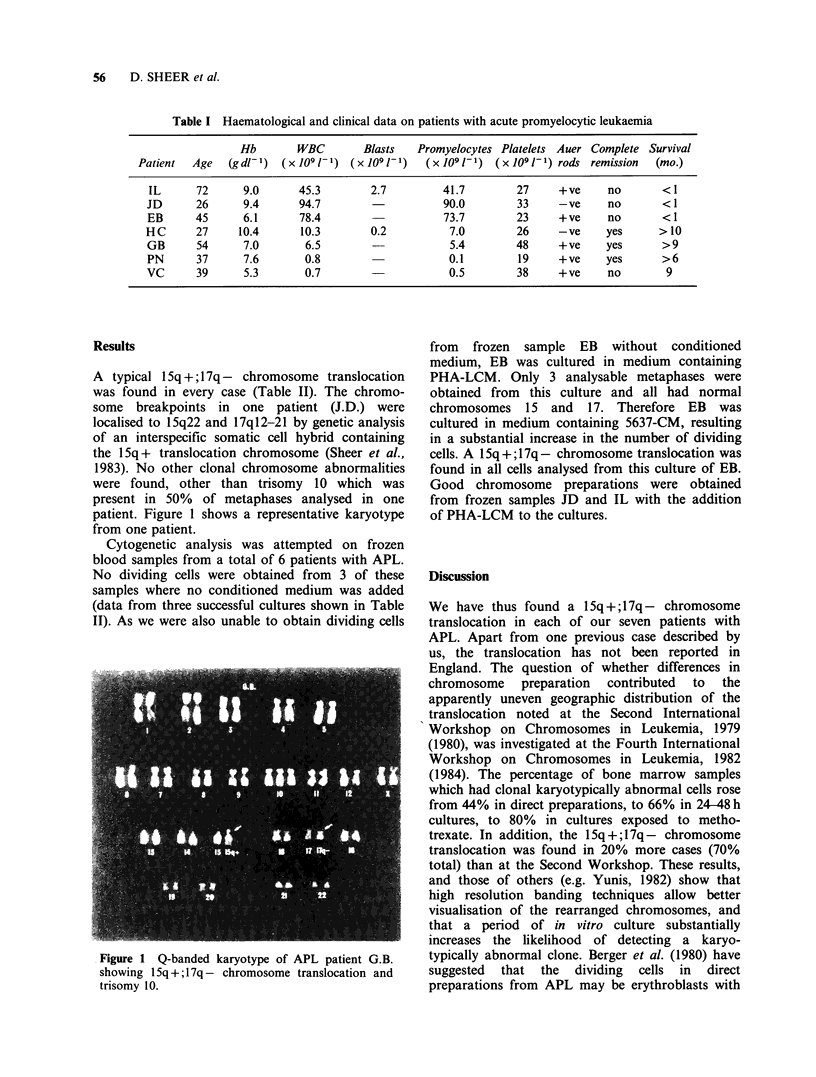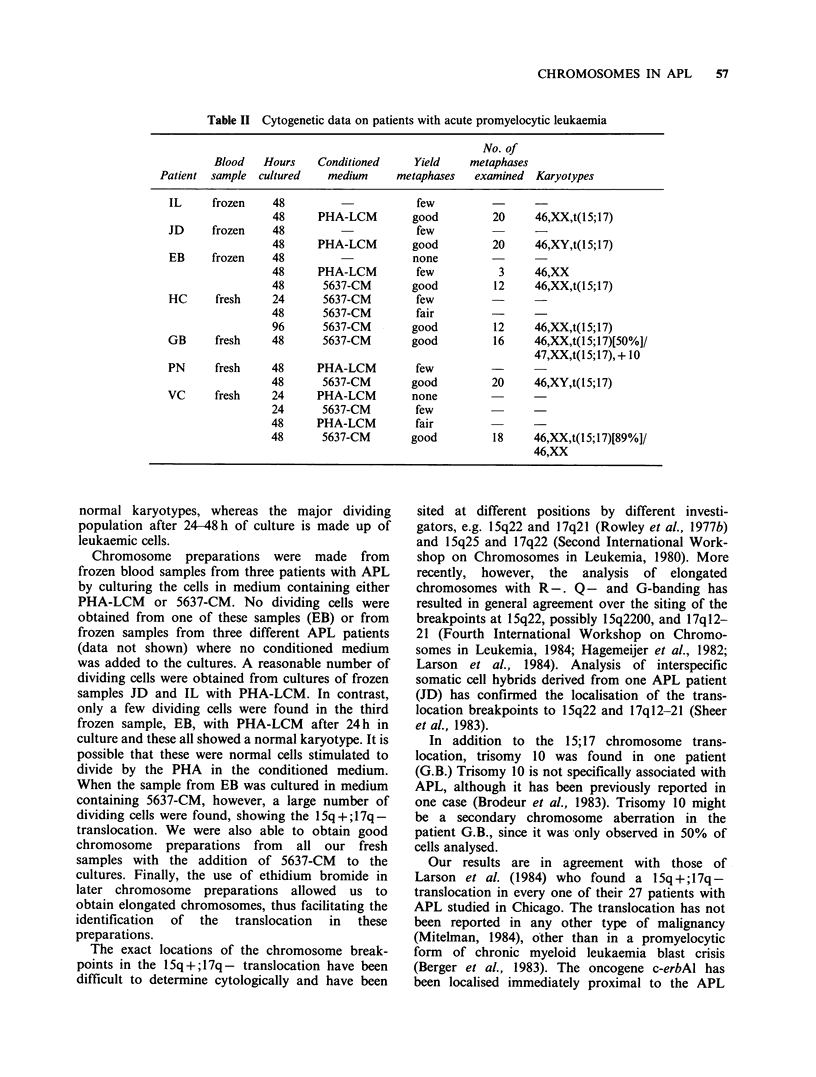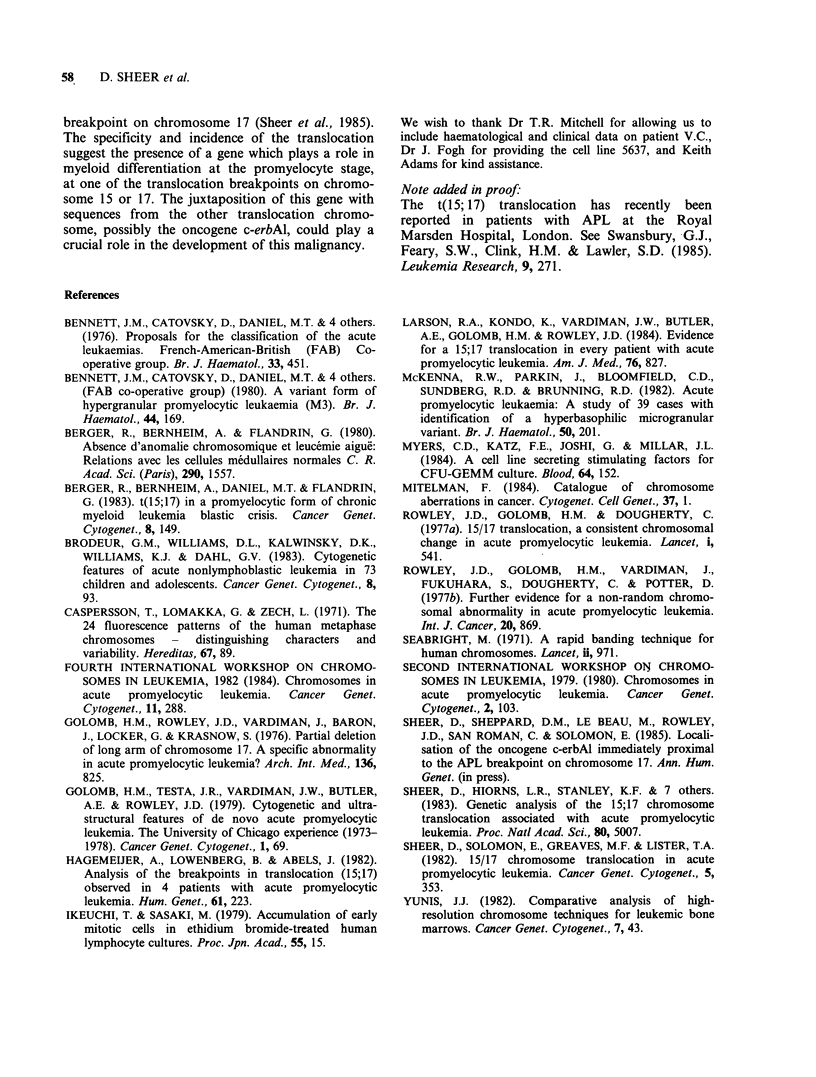# Incidence of the 15q+;17q- chromosome translocation in acute promyelocytic leukaemia (APL).

**DOI:** 10.1038/bjc.1985.148

**Published:** 1985-07

**Authors:** D. Sheer, T. A. Lister, J. Amess, E. Solomon

## Abstract

**Images:**


*Br. J. Cancer* (1985), **52**, 55–58

### Incidence of the 15q+;17q− chromosome translocation in acute promyelocytic leukaemia (APL)

D. Sheer[1], T.A. Lister[2], J. Amess[3] & E. Solomon[1]

[1]*Imperial Cancer Research Fund, P.O. Box 123, Lincoln's Inn Fields, London WC2A 3PX;* [2]*ICRF Medical Oncology Unit, and* [3]*Department of Haematology, St Bartholomew's Hospital, London EC1.*

**Summary** Cytogenetic analysis was carried out on peripheral blood cultures from seven patients with acute promyelocytic leukaemia (APL-M3). A reciprocal 15;17 chromosome translocation, t(15q+;17q−), was found in all cases, and the breakpoints estimated to be 15q22 and 17q12–21. In addition to the t(15q+;17q−), trisomy 10 was found in 50% of cells analysed in one case. These results suggest that the 15;17 chromosome translocation may be observed in most cases of APL where the leukaemic cells are cultured before cytogenetic analysis is performed. The use of conditioned media in the culture of leukaemic cells is also described.

Acute promyelocytic leukaemia (FAB-classification M3) is characterised by marked haemorrhagic episodes, disseminated intravascular coagulation, and infiltration of the bone marrow with abnormal promyelocytes containing heavy azurophilic granulation. Some promyelocytes contain multiple Auer rods (Bennett *et al.*, 1976). A morphological variant of APL has been recognised with the same clinical features as APL (called microgranular APL or M3-variant) in which the leukaemic cells have abnormal bilobed, multilobed or reniform nuclei, and are either devoid of granules or have a few fine azurophilic granules (Bennett *et al.*, 1980; McKenna *et al.*, 1982).

An abnormal chromosome 17 was first recognised in the leukaemic cells of APL patients by Golomb *et al.* (1976), and subsequently shown to result from a balanced 15q+;17q− chromosome translocation (Rowley *et al.*, 1977*a*; Golomb *et al.*, 1979). This translocation has since been identified in both the the typical and variant forms of APL, with the data collected at the Second International Workshop on Chromosomes on Leukemia, 1979 (1980) suggesting a geographic variation in the incidence 15q+;17q− chromosome translocation in APL. More recently, however, by analysing whether cells have been cultured prior to chromosome preparation, it has been shown that the apparently uneven geographic distribution can probably be accounted for by methodological differences in chromosome preparation (Berger *et al.*, 1980; Fourth International Workshop on Chromosomes in Leukemia 1982, 1984).

Four cases of APL from England were considered at the Fourth International Workshop on Chromosomes in Leukemia, 1982 (1984), and none of these showed the 15q+;17q− chromosome translocation. There has only been one report of a case of APL in England showing this translocation (Sheer *et al.*, 1982). Cytogenetic findings are now presented on a further seven patients with this disease. In addition, the use of conditioned media for obtaining chromosome preparations from frozen leukaemic cells is described.

Correspondence: D. Sheer
Received 15 February 1985; and in revised form 18 March 1985.

### Materials and methods

Patients were diagnosed as having APL (M3) on the basis of FAB criteria. Clinical details of patients at the time of diagnosis are summarised in Table I. None of the patients had received therapy prior to peripheral blood samples being taken. Peripheral blood leukocytes from three patients had been frozen in liquid nitrogen for 2–3 years prior to cytogenetic analysis. Samples were cultured for 24 to 96 h in RPMI 1640, 20% foetal calf serum, 0.03% glutamine and 20 u ml$^{-1}$ heparin. Conditioned medium from 7-day cultures of PHA-stimulated peripheral blood lymphocytes (PHA-LCM) and of the human bladder carcinoma cell line 5637 (5637-CM), which has been shown to secrete growth factors for myeloid cells (Myers *et al.*, 1984) was added to most cultures to a final concentration of 10% (Table II). Ethidium bromide was added to cultures from all patients except JD and IL, together with colcemid for 1–1.5 h prior to chromosome harvest, to a final concentration of 10 $\mu$g ml$^{-1}$ (Ikeuchi & Sasaki, 1979). Chromosomes were prepared according to standard procedures and chromosome abnormalities identified by G-banding (Seabright, 1971) and Q-banding (Caspersson *et al.*, 1971). Ten to twenty metaphase spreads were analyzed in each case.

© The Macmillan Press Ltd., 1985

**Table I** Haematological and clinical data on patients with acute promyelocytic leukaemia

| *Patient* | *Age* | *Hb* ($g\,dl^{-1}$) | *WBC* ($\times 10^9\,l^{-1}$) | *Blasts* ($\times 10^9\,l^{-1}$) | *Promyelocytes* ($\times 10^9\,l^{-1}$) | *Platelets* ($\times 10^9\,l^{-1}$) | *Auer rods* | *Complete remission* | *Survival* (*mo.*) |
|---|---|---|---|---|---|---|---|---|---|
| IL | 72 | 9.0 | 45.3 | 2.7 | 41.7 | 27 | +ve | no | <1 |
| JD | 26 | 9.4 | 94.7 | — | 90.0 | 33 | −ve | no | <1 |
| EB | 45 | 6.1 | 78.4 | — | 73.7 | 23 | +ve | no | <1 |
| HC | 27 | 10.4 | 10.3 | 0.2 | 7.0 | 26 | −ve | yes | >10 |
| GB | 54 | 7.0 | 6.5 | — | 5.4 | 48 | +ve | yes | >9 |
| PN | 37 | 7.6 | 0.8 | — | 0.1 | 19 | +ve | yes | >6 |
| VC | 39 | 5.3 | 0.7 | — | 0.5 | 38 | +ve | no | 9 |

### Results

A typical 15q+;17q− chromosome translocation was found in every case (Table II). The chromosome breakpoints in one patient (J.D.) were localised to 15q22 and 17q12–21 by genetic analysis of an interspecific somatic cell hybrid containing the 15q+ translocation chromosome (Sheer *et al.*, 1983). No other clonal chromosome abnormalities were found, other than trisomy 10 which was present in 50% of metaphases analysed in one patient. Figure 1 shows a representative karyotype from one patient.

Cytogenetic analysis was attempted on frozen blood samples from a total of 6 patients with APL. No dividing cells were obtained from 3 of these samples where no conditioned medium was added (data from three successful cultures shown in Table II). As we were also unable to obtain dividing cells from frozen sample EB without conditioned medium, EB was cultured in medium containing PHA-LCM. Only 3 analysable metaphases were obtained from this culture and all had normal chromosomes 15 and 17. Therefore EB was cultured in medium containing 5637-CM, resulting in a substantial increase in the number of dividing cells. A 15q+;17q− chromosome translocation was found in all cells analysed from this culture of EB. Good chromosome preparations were obtained from frozen samples JD and IL with the addition of PHA-LCM to the cultures.

**Figure 1** Q-banded karyotype of APL patient G.B. showing 15q+;17q− chromosome translocation and trisomy 10.

### Discussion

We have thus found a 15q+;17q− chromosome translocation in each of our seven patients with APL. Apart from one previous case described by us, the translocation has not been reported in England. The question of whether differences in chromosome preparation contributed to the apparently uneven geographic distribution of the translocation noted at the Second International Workshop on Chromosomes in Leukemia, 1979 (1980), was investigated at the Fourth International Workshop on Chromosomes in Leukemia, 1982 (1984). The percentage of bone marrow samples which had clonal karyotypically abnormal cells rose from 44% in direct preparations, to 66% in 24–48 h cultures, to 80% in cultures exposed to methotrexate. In addition, the 15q+;17q− chromosome translocation was found in 20% more cases (70% total) than at the Second Workshop. These results, and those of others (e.g. Yunis, 1982) show that high resolution banding techniques allow better visualisation of the rearranged chromosomes, and that a period of *in vitro* culture substantially increases the likelihood of detecting a karyotypically abnormal clone. Berger *et al.* (1980) have suggested that the dividing cells in direct preparations from APL may be erythroblasts with

**Table II** Cytogenetic data on patients with acute promyelocytic leukaemia

| *Patient* | *Blood sample* | *Hours cultured* | *Conditioned medium* | *Yield metaphases* | *No. of metaphases examined* | *Karyotypes* |
|---|---|---|---|---|---|---|
| IL | frozen | 48 | — | few | — | — |
| | | 48 | PHA-LCM | good | 20 | 46,XX,t(15;17) |
| JD | frozen | 48 | — | few | — | — |
| | | 48 | PHA-LCM | good | 20 | 46,XY,t(15;17) |
| EB | frozen | 48 | — | none | — | — |
| | | 48 | PHA-LCM | few | 3 | 46,XX |
| | | 48 | 5637-CM | good | 12 | 46,XX,t(15;17) |
| HC | fresh | 24 | 5637-CM | few | — | — |
| | | 48 | 5637-CM | fair | — | — |
| | | 96 | 5637-CM | good | 12 | 46,XX,t(15;17) |
| GB | fresh | 48 | 5637-CM | good | 16 | 46,XX,t(15;17)[50%]/ 47,XX,t(15;17),+10 |
| PN | fresh | 48 | PHA-LCM | few | — | — |
| | | 48 | 5637-CM | good | 20 | 46,XY,t(15;17) |
| VC | fresh | 24 | PHA-LCM | none | — | — |
| | | 24 | 5637-CM | few | — | — |
| | | 48 | PHA-LCM | fair | — | — |
| | | 48 | 5637-CM | good | 18 | 46,XX,t(15;17)[89%]/ 46,XX |

normal karyotypes, whereas the major dividing population after 24–48 h of culture is made up of leukaemic cells.

Chromosome preparations were made from frozen blood samples from three patients with APL by culturing the cells in medium containing either PHA-LCM or 5637-CM. No dividing cells were obtained from one of these samples (EB) or from frozen samples from three different APL patients (data not shown) where no conditioned medium was added to the cultures. A reasonable number of dividing cells were obtained from cultures of frozen samples JD and IL with PHA-LCM. In contrast, only a few dividing cells were found in the third frozen sample, EB, with PHA-LCM after 24 h in culture and these all showed a normal karyotype. It is possible that these were normal cells stimulated to divide by the PHA in the conditioned medium. When the sample from EB was cultured in medium containing 5637-CM, however, a large number of dividing cells were found, showing the 15q+;17q− translocation. We were also able to obtain good chromosome preparations from all our fresh samples with the addition of 5637-CM to the cultures. Finally, the use of ethidium bromide in later chromosome preparations allowed us to obtain elongated chromosomes, thus facilitating the identification of the translocation in these preparations.

The exact locations of the chromosome breakpoints in the 15q+;17q− translocation have been difficult to determine cytologically and have been sited at different positions by different investigators, e.g. 15q22 and 17q21 (Rowley *et al.*, 1977*b*) and 15q25 and 17q22 (Second International Workshop on Chromosomes in Leukemia, 1980). More recently, however, the analysis of elongated chromosomes with R−. Q− and G-banding has resulted in general agreement over the siting of the breakpoints at 15q22, possibly 15q2200, and 17q12–21 (Fourth International Workshop on Chromosomes in Leukemia, 1984; Hagemeijer *et al.*, 1982; Larson *et al.*, 1984). Analysis of interspecific somatic cell hybrids derived from one APL patient (JD) has confirmed the localisation of the translocation breakpoints to 15q22 and 17q12–21 (Sheer *et al.*, 1983).

In addition to the 15;17 chromosome translocation, trisomy 10 was found in one patient (G.B.) Trisomy 10 is not specifically associated with APL, although it has been previously reported in one case (Brodeur *et al.*, 1983). Trisomy 10 might be a secondary chromosome aberration in the patient G.B., since it was only observed in 50% of cells analysed.

Our results are in agreement with those of Larson *et al.* (1984) who found a 15q+;17q− translocation in every one of their 27 patients with APL studied in Chicago. The translocation has not been reported in any other type of malignancy (Mitelman, 1984), other than in a promyelocytic form of chronic myeloid leukaemia blast crisis (Berger *et al.*, 1983). The oncogene c-*erb*A1 has been localised immediately proximal to the APL

breakpoint on chromosome 17 (Sheer *et al.*, 1985). The specificity and incidence of the translocation suggest the presence of a gene which plays a role in myeloid differentiation at the promyelocyte stage, at one of the translocation breakpoints on chromosome 15 or 17. The juxtaposition of this gene with sequences from the other translocation chromosome, possibly the oncogene c-*erb*Al, could play a crucial role in the development of this malignancy.

We wish to thank Dr T.R. Mitchell for allowing us to include haematological and clinical data on patient V.C., Dr J. Fogh for providing the cell line 5637, and Keith Adams for kind assistance.

*Note added in proof:*
The t(15; 17) translocation has recently been reported in patients with APL at the Royal Marsden Hospital, London. See Swansbury, G.J., Feary, S.W., Clink, H.M. & Lawler, S.D. (1985). *Leukemia Research*, **9**, 271.

### References

BENNETT, J.M., CATOVSKY, D., DANIEL, M.T. & 4 others. (1976). Proposals for the classification of the acute leukaemias. French-American-British (FAB) Co-operative group. *Br. J. Haematol.*, **33**, 451.

BENNETT, J.M., CATOVSKY, D., DANIEL, M.T. & 4 others. (FAB co-operative group) (1980). A variant form of hypergranular promyelocytic leukaemia (M3). *Br. J. Haematol.*, **44**, 169.

BERGER, R., BERNHEIM, A. & FLANDRIN, G. (1980). Absence d'anomalie chromosomique et leucémie aiguë: Relations avec les cellules médullaires normales *C. R. Acad. Sci. (Paris)*, **290**, 1557.

BERGER, R., BERNHEIM, A., DANIEL, M.T. & FLANDRIN, G. (1983). t(15;17) in a promyelocytic form of chronic myeloid leukemia blastic crisis. *Cancer Genet. Cytogenet.*, **8**, 149.

BRODEUR, G.M., WILLIAMS, D.L., KALWINSKY, D.K., WILLIAMS, K.J. & DAHL, G.V. (1983). Cytogenetic features of acute nonlymphoblastic leukemia in 73 children and adolescents. *Cancer Genet. Cytogenet.*, **8**, 93.

CASPERSSON, T., LOMAKKA, G. & ZECH, L. (1971). The 24 fluorescence patterns of the human metaphase chromosomes – distinguishing characters and variability. *Hereditas*, **67**, 89.

FOURTH INTERNATIONAL WORKSHOP ON CHROMOSOMES IN LEUKEMIA, 1982 (1984). Chromosomes in acute promyelocytic leukemia. *Cancer Genet. Cytogenet.*, **11**, 288.

GOLOMB, H.M., ROWLEY, J.D., VARDIMAN, J., BARON, J., LOCKER, G. & KRASNOW, S. (1976). Partial deletion of long arm of chromosome 17. A specific abnormality in acute promyelocytic leukemia? *Arch. Int. Med.*, **136**, 825.

GOLOMB, H.M., TESTA, J.R., VARDIMAN, J.W., BUTLER, A.E. & ROWLEY, J.D. (1979). Cytogenetic and ultrastructural features of de novo acute promyelocytic leukemia. The University of Chicago experience (1973–1978). *Cancer Genet. Cytogenet.*, **1**, 69.

HAGEMEIJER, A., LOWENBERG, B. & ABELS, J. (1982). Analysis of the breakpoints in translocation (15;17) observed in 4 patients with acute promyelocytic leukemia. *Hum. Genet.*, **61**, 223.

IKEUCHI, T. & SASAKI, M. (1979). Accumulation of early mitotic cells in ethidium bromide-treated human lymphocyte cultures. *Proc. Jpn. Acad.*, **55**, 15.

LARSON, R.A., KONDO, K., VARDIMAN, J.W., BUTLER, A.E., GOLOMB, H.M. & ROWLEY, J.D. (1984). Evidence for a 15;17 translocation in every patient with acute promyelocytic leukemia. *Am. J. Med.*, **76**, 827.

McKENNA, R.W., PARKIN, J., BLOOMFIELD, C.D., SUNDBERG, R.D. & BRUNNING, R.D. (1982). Acute promyelocytic leukaemia: A study of 39 cases with identification of a hyperbasophilic microgranular variant. *Br. J. Haematol.*, **50**, 201.

MYERS, C.D., KATZ, F.E., JOSHI, G. & MILLAR, J.L. (1984). A cell line secreting stimulating factors for CFU-GEMM culture. *Blood*, **64**, 152.

MITELMAN, F. (1984). Catalogue of chromosome aberrations in cancer. *Cytogenet. Cell Genet.*, **37**, 1.

ROWLEY, J.D., GOLOMB, H.M. & DOUGHERTY, C. (1977*a*). 15/17 translocation, a consistent chromosomal change in acute promyelocytic leukemia. *Lancet*, **i**, 541.

ROWLEY, J.D., GOLOMB, H.M., VARDIMAN, J., FUKUHARA, S., DOUGHERTY, C. & POTTER, D. (1977*b*). Further evidence for a non-random chromosomal abnormality in acute promyelocytic leukemia. *Int. J. Cancer*, **20**, 869.

SEABRIGHT, M. (1971). A rapid banding technique for human chromosomes. *Lancet*, **ii**, 971.

SECOND INTERNATIONAL WORKSHOP ON CHROMOSOMES IN LEUKEMIA, 1979. (1980). Chromosomes in acute promyelocytic leukemia. *Cancer Genet. Cytogenet.*, **2**, 103.

SHEER, D., SHEPPARD, D.M., LE BEAU, M., ROWLEY, J.D., SAN ROMAN, C. & SOLOMON, E. (1985). Localisation of the oncogene c-erbAl immediately proximal to the APL breakpoint on chromosome 17. *Ann. Hum. Genet.* (in press).

SHEER, D., HIORNS, L.R., STANLEY, K.F. & 7 others. (1983). Genetic analysis of the 15;17 chromosome translocation associated with acute promyelocytic leukemia. *Proc. Natl Acad. Sci.*, **80**, 5007.

SHEER, D., SOLOMON, E., GREAVES, M.F. & LISTER, T.A. (1982). 15/17 chromosome translocation in acute promyelocytic leukemia. *Cancer Genet. Cytogenet.*, **5**, 353.

YUNIS, J.J. (1982). Comparative analysis of high-resolution chromosome techniques for leukemic bone marrows. *Cancer Genet. Cytogenet.*, **7**, 43.